# Editorial: Telomeres and Epigenetics in Endocrinology

**DOI:** 10.3389/fendo.2019.00257

**Published:** 2019-04-24

**Authors:** Yiqiang Zhan, Huan Song

**Affiliations:** ^1^Department of Medical Epidemiology and Biostatistics, Karolinska Institutet, Stockholm, Sweden; ^2^Center of Public Health Sciences, University of Iceland, Reykjavík, Iceland

**Keywords:** telomeres, epigenetics, endocrinology, aging, risk factors, prediction, diagnosis

Aging, the multifactorial process in which a progressive decline at the cellular, tissue, and organismal levels leads to death, has perpetually fascinated human beings around the world. In addition to the apparent signs of aging, such as gray hair, skin wrinkling, and muscle atrophy, other potential biological markers that are of predictive values still deserve to be determined. The most recent efforts on this topic have discovered two biomarkers that are of significance in the aging research field: telomeres and epigenetics (1).

Telomeres are a repetitive sequence of nucleotides (TTAGGG) at the end of chromosomes. Since being discovered, they have been hypothesized as the fundamental aspect of cellular senescence. In particular, the length of telomeres gradually decreases as cells divide. In humans, the length of telomeres is ~5,000–10,000 base pairs for newborns, which begins to decline at the rate of 20–40 base pairs/year. Accumulating evidence has found that shorter telomeres were associated with cardiovascular diseases (2–6), diabetes (7), stroke (8), dementia and brain health (9–12), and mortality (13, 14). However, the evidence for the roles of telomeres in endocrinology is not conclusive. For example, it is not clear if some biomarkers of endocrinology, such as vitamin D, peptides, insulin, and diabetes, and other glycemic traits are associated with telomeres and what the potential implications of these biomarkers and telomeres in the clinics of endocrinology and other clinical departments are. The same questions could also be applied to epigenetics, another aging biomarker. Epigenetics primarily represents the changes that affect the activity and expression of genes. These changes can result from environmental factors, such as smoking behavior and other exposures. Of note, is that epigenetic changes have been found to be associated with aging-related outcomes, and are also involved in endocrinology. However, more evidence is needed, and further elucidation of the biological mechanisms is warranted. With these knowledge gaps, we initiated the special topic collection and attracted these articles in the field of endocrinology of aging.

In the first article, Yang et al. reported a study that examined the relationship of telomeres with C-peptide (Yang et al.) and found a negative/inverse association between longer telomeres and lower levels of C-peptide. C-peptide is a short polypeptide with 31-amino-acid and relates insulin's A-chain to its B-chain in the proinsulin molecule. It was first described in connection with the discovery of the insulin biosynthesis pathway half a century ago. In clinical settings, patients with diabetes may have their C-peptide levels measured to distinguish type 1 diabetes and type 2 diabetes. The study performed by Yang et al. made multivariate adjustments and controlled for a few other factors, including age and sex, and thus offered new evidence to support that telomeres are associated with glycemic traits, albeit with a small magnitude.

The second article by Pavanello et al. presented a study that examined the interaction between obesity and locus at *CYP27A1* (encoding sterol 27-hydroxylase, a largely distributed mitochondrial P450 cytochrome enzyme that converts extrahepatic cholesterol to 27-hydroxycholesterol) and their associations with cardio-metabolic risk factors and telomeres (Provenzi et al.). The authors found that telomeres were shortened in obese participants with more than three risk alleles of *CYP27A1*, and suggested that the role of *CYP27A1* on the relationship between obesity and telomeres provided further insights into mechanisms linking obesity with the biology of aging. This study was hypothesis-driven; the genetic variants were selected for being previously and substantially genotyped. The big sample size and the rich panel of other biomarkers allowed the authors to conduct much more detailed analyses on this topic.

The third article by Provenzi et al. proposed their perspectives on the role of telomeres in premature birth and discussed the potential implications for early adversity and care in the neonatal intensive care unit (Pavanello et al.). Indeed, the speculation of telomeres in aging begins in the premature aging syndrome. It is thus interesting to examine if telomeres also play a role in premature birth/infants. The perspectives presented by Provenzi et al. are of interest in both theory and clinical practice and definitely need to be investigated further in the future.

The last article by Lin et al. studied dietary copper intake and its relation with telomeres. The authors found that in this study sample, telomeres were longer for people with more dietary copper intake (Lin et al.). The significant positive association did not change substantially in the multivariate analyses. Copper is one of the essential trace elements and major components for enzymes in humans and other species. While the proposed research topic is interesting, there is not too much evidence to support the speculation as noted by the authors. As dietary copper intake differs from environmental copper exposure, the study by Lin et al. may offer novel insights into the biological benefits and/or mechanisms of dietary or even circulating copper in human health.

In summary, we assembled articles from diverse researchers in the field of telomeres and aging. It is a pity that we did not have articles on epigenetics. The articles collected so far could add new knowledge, evidence, and perspectives on the roles of telomeres in endocrinology.

## Author Contributions

All authors listed have made a substantial, direct and intellectual contribution to the work, and approved it for publication.

### Conflict of Interest Statement

The authors declare that the research was conducted in the absence of any commercial or financial relationships that could be construed as a potential conflict of interest.
